# Probing auditory scene analysis

**DOI:** 10.3389/fnins.2014.00293

**Published:** 2014-09-12

**Authors:** Susann Deike, Susan L. Denham, Elyse Sussman

**Affiliations:** ^1^Special Lab Non-Invasive Brain Imaging, Leibniz Institute for NeurobiologyMagdeburg, Germany; ^2^Cognition Institute, University of PlymouthPlymouth, UK; ^3^School of Psychology, University of PlymouthPlymouth, UK; ^4^Department of Neuroscience, Albert Einstein College of Medicine of Yeshiva UniversityBronx, NY, USA; ^5^Department of Otorhinolaryngology-Head and Neck Surgery, Albert Einstein College of Medicine of Yeshiva UniversityBronx, NY, USA

**Keywords:** auditory scene analysis, multistable perception, ambiguity, realistic auditory scenes, stream segregation

In natural environments, the auditory system is typically confronted with a mixture of sounds originating from different sound sources. The sounds emanating from different sources can overlap each other in time and feature space. Thus, the auditory system has to continuously decompose competing sounds into distinct meaningful auditory objects or “auditory streams” associated with the possible sound sources. This decomposition of the sounds, termed “Auditory scene analysis” (ASA) by Bregman (1990), involves two kinds of grouping. Grouping based on simultaneous cues (e.g., harmonicity) and on sequential cues (e.g., similarity of acoustic features over time). Understanding how the brain solves these tasks is a fundamental challenge facing auditory scientists. In recent years, the topic of ASA was broadly investigated in different fields of auditory research using a wide range of methods, including studies in different species (Hulse et al., 1997; Fay, 2000; Fishman et al., 2001; Moss and Surlykke, 2001), and computer modeling of ASA (for recent reviews see, Winkler et al., 2012; Gutschalk and Dykstra, 2014). Despite advances in understanding ASA, it still proves to be a major challenge for auditory research, especially in verifying whether experimental findings are transferable to more realistic auditory scenes. This special issue is a collection of 10 research papers and one review paper providing a snapshot of current ASA research. The research paper on visual perception provides a comparative view of modality specific as well as general characteristics of perception.

One approach for understanding ASA in real auditory scenes is the use of stimulus parameters that produce an ambiguous percept (cf. Pressnitzer et al., 2011). The advantage of such an approach is that different perceptual organizations can be studied without varying physical stimulus parameters. Using a visual ambiguous stimulus and combining real-time functional magnetic resonance imaging and machine learning techniques, Reichert et al. (2014) showed that it is possible to determine the momentary state of a subject's conscious percept from time resolved BOLD-activity. The high classification accuracy of this data-driven classification approach may be particularly useful for auditory research investigating perception in continuous, ecologically-relevant sound scenes.

A second advantage in using ambiguous stimuli in experiments on ASA is that perception of them can be influenced by intention or task (Moore and Gockel, 2002). By manipulating task requirements one can mirror real hearing situations where listeners often need to identify and localize sound sources. The studies by Shestopalova et al. (2014) and Kondo et al. (2014) examined the influence of motion on stream segregation. In general, and corresponding to earlier findings, both of these studies found that sound source separation in space promoted segregation. Surprisingly, however, the effect of spatial separation on stream segregation was found to be temporally limited and affected by volitional head motion (Kondo et al., 2014), but unaffected by movement of sound sources or by the presentation of movement-congruent visual cues (Shestopalova et al., 2014). Another study, by Sussman-Fort and Sussman (2014), investigated the influence of stimulus context on the buildup of stream segregation. They found that the build-up of stream segregation was context-dependent, occurring faster under constant than varying stimulus conditions. Based on these findings the authors suggested that the auditory system maintains a representation of the environment that is only updated when new information indicates that reanalyzing the scene is necessary. Two further studies examined the influence of attention on stream segregation. Nie et al. (2014) found that in conditions of weak spectral contrast, attention facilitated stream segregation. Shuai and Elhilali (2014) found that different forms of attention, both stimulus-driven and top-down attentional processes, modulated the response to a salient event detected within a sound stream.

The special issue also includes two research papers that extend current views on multistability and perceptual ambiguity. The psychophysical study by Denham et al. (2014) showed that streaming sequences could be perceived in many more ways than in the traditionally assumed (Integrated vs. Segregated organizations) and that the different interpretations continuously compete for dominance. Moreover, despite being highly stochastic, the switching patterns of individual participants could be distinguished from those of others. Hence, perceptual multistability can be used to characterize both general mechanisms and individual differences in human perception. By comparing stimulus conditions that promote one perceptual organization with those causing an ambiguous percept Dollezal et al. (2014) found specific BOLD responses for the ambiguous condition in higher cognitive areas (i.e., posterior medial prefrontal cortex and posterior cingulate cortex). Both of these regions were associated with cognitive functions, monitoring decision uncertainty (Ridderinkhof et al., 2004) and being involved when higher task demands were imposed (Raichle et al., 2001; Dosenbach et al., 2007), respectively. This suggests that perceptual ambiguity may be characterized by uncertainty regarding the appropriate perceptual organization, and by higher cognitive load due to this uncertainty.

A second group of research papers within this special issue focused on understanding hearing deficits in older listeners and cochlear implant (CI) users. Gallun et al. (2013) demonstrated that listeners could be categorized in terms of their ability to use spatial and spectrotemporal cues to separate competing speech streams. They showed that the factor of age substantially reduced spatial release from masking, supporting the hypothesis that aging, independent of an individual's hearing threshold, can result in changes in the cortical and/or subcortical structures essential for spatial hearing. Divenyi (2014) compared the signal to noise (S/N) ratio at which normal hearing young and elderly listeners were able to discriminate single formant dynamics in vowel-analog streams and found that elderly listeners required a 15 and 20 dB larger S/N ratio than younger listeners. Since formant transitions represent potent cues for speech intelligibility, this result may at least partially explain the well-documented intelligibility loss of speech in babble noise by the elderly. Böckmann-Barthel et al. (2014) pursued the question whether the time course of auditory streaming differs between normal-hearing listeners and CI users and found that the perception of streaming sequences was similar in quality between both groups. This similarity may suggest that stream segregation is not solely determined by frequency discrimination, and that CI users do not simply respond to differences between A and B sounds but actually experience the phenomenon of stream segregation.

The review by Bendixen (2014) suggests predictability as a cue for sound source decomposition. Bendixen collected empirical evidence spanning issues of predictive auditory processing, predictive processing in ASA, and methodological aspects of measuring ASA. As a result, and as a theoretical framework, an analogy with the old-plus-new heuristic for grouping simultaneous acoustic signals was proposed.

Taken together, this special issue provides a comprehensive summary of current research in ASA, relating the approaches and experimental findings to natural listening conditions. It would be highly desirable in future research on ASA to use more natural stimuli and to test the ecological validity of these findings. With this special issue we hope to raise awareness of this issue.

## Conflict of interest statement

The authors declare that the research was conducted in the absence of any commercial or financial relationships that could be construed as a potential conflict of interest.

## References

[B1] Bendixen A. (2014). Predictability effects in auditory scene analysis: a review. Front. Neurosci. 8:60 10.3389/fnins.2014.00060 24744695 PMC3978260

[B2] Böckmann-Barthel M. Deike S. Brechmann A. Ziese M. Verhey J. L. (2014). Time course of auditory streaming: do CI users differ from normal-hearing listeners? Front. Psychol. 5:775 10.3389/fpsyg.2014.00775 25101035 PMC4104638

[B3] Bregman A. S. (1990). Auditory Scene Analysis. The Perceptual Organization of Sound. Cambridge: MIT Press

[B4] Denham S. Bohm T. M. Bendixen A. Szalardy O. Kocsis Z. Mill R. (2014). Stable individual characteristics in the perception of multiple embedded patterns in multistable auditory stimuli. Front. Neurosci. 8:25 10.3389/fnins.2014.00025 24616656 PMC3937586

[B5] Divenyi P. (2014). Decreased ability in the segregation of dynamically changing vowel-analog streams: a factor in the age-related cocktail-party deficit? Front. Neurosci. 8:144 10.3389/fnins.2014.00144 24971047 PMC4054799

[B6] Dollezal L. V. Brechmann A. Klump G. M. Deike S. (2014). Evaluating auditory stream segregation of SAM tone sequences by subjective and objective psychoacoustical tasks, and brain activity. Front. Neurosci. 8:119 10.3389/fnins.2014.00119 24936170 PMC4047832

[B7] Dosenbach N. U. Fair D. A. Miezin F. M. Cohen A. L. Wenger K. K. Dosenbach R. A. (2007). Distinct brain networks for adaptive and stable task control in humans. Proc. Natl. Acad. Sci. U.S.A. 104, 11073–11078 10.1073/pnas.0704320104 17576922 PMC1904171

[B7a] Fay R. R. (2000). Spectral contrasts underlying auditory stream segregation in goldfish (*Carassius auratus*). J. Assoc. Res. Otolaryngol. 1, 120–128 10.1007/s101620010015 11545140 PMC2504535

[B7b] Fishman Y. I. Reser D. H. Arezzo J. C. Steinschneider M. (2001). Neural correlates of auditory stream segregation in primary auditory cortex of the awake monkey. Hear. Res. 151, 167–187 10.1016/S0378-5955(00)00224-0 11124464

[B8] Gallun F. J. Diedesch A. C. Kampel S. D. Jakien K. M. (2013). Independent impacts of age and hearing loss on spatial release in a complex auditory environment. Front. Neurosci. 7:252 10.3389/fnins.2013.00252 24391535 PMC3870327

[B9] Gutschalk A. Dykstra A. R. (2014). Functional imaging of auditory scene analysis. Hear. Res. 307, 98–110 10.1016/j.heares.2013.08.003 23968821

[B9a] Hulse S. H. MacDougall-Shackleton S. A. Wisniewski A. B. (1997). Auditory scene analysis by songbirds: stream segregation of birdsong by European starlings (*Sturnus vulgaris*). J. Comp. Psychol. 111, 3–13 10.1037/0735-7036.111.1.3 9090135

[B10] Kondo H. M. Toshima I. Pressnitzer D. Kashino M. (2014). Probing the time course of head-motion cues integration during auditory scene analysis. Front. Neurosci. 8:170 10.3389/fnins.2014.00170 25009456 PMC4067593

[B11] Moore B. C. Gockel H. (2002). Factors influencing sequential stream segregation. Acta Acustica United with Acustica 88, 320–332

[B11a] Moss C. F. Surlykke A. (2001). Auditory scene analysis by echolocation in bats. J. Acoust. Soc. Am. 110, 2207–2226 10.1121/1.1398051 11681397

[B12] Nie Y. Zhang Y. Nelson P. B. (2014). Auditory stream segregation using bandpass noises: evidence from event-related potentials. Front. Neurosci. 8:277 10.3389/fnins.2014.00277 PMC4162371 25309306

[B13] Pressnitzer D. Suied C. Shamma S. A. (2011). Auditory scene analysis: the sweet music of ambiguity. Front. Hum. Neurosci. 5:158 10.3389/fnhum.2011.00158 22174701 PMC3237025

[B14] Raichle M. E. Macleod A. M. Snyder A. Z. Powers W. J. Gusnard D. A. Shulman G. L. (2001). A default mode of brain function. Proc. Natl. Acad. Sci. U.S.A. 98, 676–682 10.1073/pnas.98.2.676 11209064 PMC14647

[B15] Reichert C. Fendrich R. Bernarding J. Tempelmann C. Hinrichs H. Rieger J. W. (2014). Online tracking of the contents of conscious perception using real-time fMRI. Front. Neurosci. 8:116 10.3389/fnins.2014.00116 24904260 PMC4033165

[B16] Ridderinkhof K. R. Ullsperger M. Crone E. A. Nieuwenhuis S. (2004). The role of the medial frontal cortex in cognitive control. Science 306, 443–447 10.1126/science.1100301 15486290

[B17] Shestopalova L. Bohm T. M. Bendixen A. Andreou A. G. Georgiou J. Garreau G. (2014). Do audio-visual motion cues promote segregation of auditory streams? Front. Neurosci. 8:64 10.3389/fnins.2014.00064 24778604 PMC3985028

[B18] Shuai L. Elhilali M. (2014). Task-dependent neural representations of salient events in dynamic auditory scenes. Front. Neurosci. 8:203 10.3389/fnins.2014.00203 25100934 PMC4104552

[B19] Sussman-Fort J. Sussman E. (2014). The effect of stimulus context on the buildup to stream segregation. Front. Neurosci. 8:93 10.3389/fnins.2014.00093 24808822 PMC4010780

[B20] Winkler I. Denham S. Mill R. Bohm T. M. Bendixen A. (2012). Multistability in auditory stream segregation: a predictive coding view. Philos. Trans. R. Soc. Lond. B Biol. Sci. 367, 1001–1012 10.1098/rstb.2011.0359 22371621 PMC3282310

