# Corrigendum: Probing auditory scene analysis

**DOI:** 10.3389/fnins.2014.00367

**Published:** 2014-11-13

**Authors:** Susann Deike, Susan L. Denham, Elyse S. Sussman

**Affiliations:** ^1^Special Lab Non-invasive Brain Imaging, Leibniz Institute for NeurobiologyMagdeburg, Germany; ^2^Cognition Institute, University of PlymouthPlymouth, UK; ^3^School of Psychology, University of PlymouthPlymouth, UK; ^4^Department of Neuroscience, Albert Einstein College of MedicineBronx, NY, USA; ^5^Department of Otorhinolaryngology-Head and Neck Surgery, Albert Einstein College of MedicineBronx, NY, USA

**Keywords:** auditory scene analysis, multistable perception, ambiguity, realistic auditory scenes, stream segregation

One of the funding sources was omitted from the Acknowledgments list and one funding source was incorrectly assigned. The corrected list is as follows. We thank the authors for their contributions and the reviewers for their useful comments. Susann Deike was funded by the “Deutsche Forschungsgemeinschaft” [SFB/TRR31]. Elyse S. Sussman was funded by the National Institutes of Health (DC004263).

## Conflict of interest statement

The authors declare that the research was conducted in the absence of any commercial or financial relationships that could be construed as a potential conflict of interest.

